# Formal Semantics and Verification of Network-Based Biocomputation Circuits

**DOI:** 10.1007/978-3-030-67067-2_21

**Published:** 2020-12-15

**Authors:** Michelle Aluf-Medina, Till Korten, Avraham Raviv, Dan V. Nicolau Jr., Hillel Kugler

**Affiliations:** 8 University of Copenhagen, Copenhagen, Denmark; 9 Tel Aviv University, Tel Aviv, Israel; 10 Technion, Haifa, Israel; 11 Bar-Ilan University, Ramat Gan, Israel; 12 B CUBE - Center for Molecular Bioengineering, TU Dresden, Dresden, Germany; 13 QUT, Brisbane, Australia

**Keywords:** Biological computation, Network-based biocomputation, Model checking, Subset sum problem, Exact cover, Satisfiability

## Abstract

Network-Based Biocomputation Circuits (NBCs) offer a new paradigm for solving complex computational problems by utilizing biological agents that operate in parallel to explore manufactured planar devices. The approach can also have future applications in diagnostics and medicine by combining NBCs computational power with the ability to interface with biological material. To realize this potential, devices should be designed in a way that ensures their correctness and robust operation. For this purpose, formal methods and tools can offer significant advantages by allowing investigation of design limitations and detection of errors before manufacturing and experimentation. Here we define a computational model for NBCs by providing formal semantics to NBC circuits. We present a formal verification-based approach and prototype tool that can assist in the design of NBCs by enabling verification of a given design’s correctness. Our tool allows verification of the correctness of NBC designs for several NP-Complete problems, including the Subset Sum, Exact Cover and Satisfiability problems and can be extended to other NBC implementations. Our approach is based on defining transition systems for NBCs and using temporal logic for specifying and proving properties of the design using model checking. Our formal model can also serve as a starting point for computational complexity studies of the power and limitations of NBC systems.

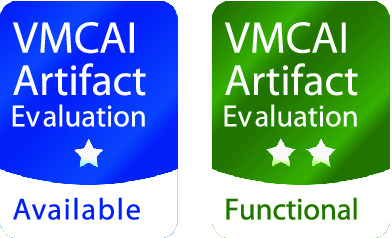



## Introduction

Engineering biological devices to perform computation is of major interest due to the potential of utilizing inherent parallelism in biological components to speed up computation, construct low energy consuming devices and interface with biological material, opening up potential diagnostic and medical applications. Network-Based Biocomputation Circuits (NBCs) [4, 20] offer a new paradigm for solving complex computational problems by utilizing biological agents that operate in parallel to explore manufactured planar devices. Devices should be designed to ensure correctness and robust operation, for which formal reasoning tools can offer significant advantages by assisting in identification of limitations and errors in the design before device manufacturing. Here we define a computational model for NBCs [20] by providing formal semantics, and present a formal verification-based approach and tool that can prove correctness of the design. The tool can be used to verify that a given design contains no logical errors, and allows evaluation of different designs prior to manufacturing. Similar verification tools are now commonplace in the hardware industry, where early identification of design flaws can lead to significant savings in cost (money, development time and reputation).

NBC is an alternative parallel-computation method that was proposed in [20] and solves a given combinatorial problem by encoding it into a graphical, molecular network that is embedded in a nanofabricated planar device. The approach can be applied for solving NP-Complete problems [14] and other types of combinatorial problems. In addition, since biological agents are utilized in NBC, the technology can be used in the future to carry cells through the devices and perform complex computational processing with medical and diagnostic applications. In the NBC approach a device runs biological agents through the network in order to explore it in parallel and thus solve a given combinatorial problem. The combinatorial problem considered in [20] is the Subset Sum Problem (SSP), which is a known NP-complete problem. The SSP problem is given a target goal *k*, and asks if it can be reached as a sum of some combination of elements in a given set $$S = \begin{Bmatrix} s_1&s_2&\ldots&s_N\end{Bmatrix}$$.

**Fig. 1.**
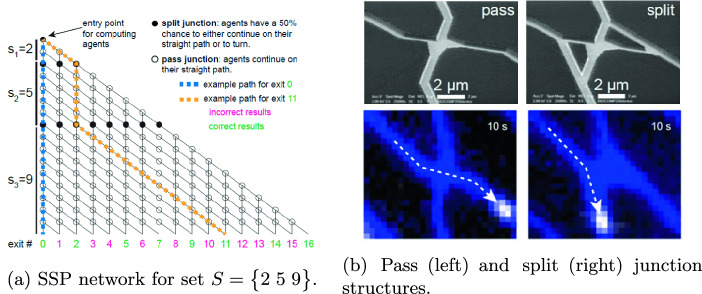
Network design for SSP (reproduced from [20]). (a) Overall network structure of the SSP for the set $$S = \begin{Bmatrix} 2&5&9 \end{Bmatrix}$$. Split junctions are denoted as filled black circles and pass junctions as unfilled circles. Agents enter from the top left point of the network. The yellow path corresponds to the sum 11 being computed utilizing 2 and 9. (b) Physical design of pass and split junctions. Pass junctions are designed to maintain the agent’s direction of movement, while split junctions are designed to allow agents an approximately equal chance to maintain or change their direction of movement.

An example NBC circuit for the SSP of $$S = \begin{Bmatrix} 2&5&9\end{Bmatrix}$$ is shown in Fig. 1a. Molecular agents (actin filaments or microtubules, which are propelled by molecular motors) enter from the top-left corner of the network. At split junctions, the agents have an approximately equal chance of moving down or moving diagonally, while agents continue in the current direction of movement at pass junctions, as seen in Fig. 1b. When a computational agent takes the diagonal path at a split junction, the element for that junction is “added”. Agents exiting the network in the bottom row thus have an *x* coordinate (denoted exit$\#$ in Fig. 1a) that represents a possible subset sum, and by utilizing many agents to explore the network in parallel all the possible subset sums can be determined.

More recently, the NBC approach has been extended to encode and solve additional NP-Complete problems [16, 32] and work has been done towards improving the scalability of the approach and the design process of the circuits. New encodings include the Exact Cover (ExCov) and the Satisfiability (SAT) problems. An additional feature that could extend the capabilities of NBC is tagging—the ability to mark a protein or filament with a distinguishing attribute. Fluorescence tagging, for example, is common in biological research and is used to track biomolecules and cells. As an additional component of computation, tagging can be used to track the paths used by computational agents [20, 27]. Once the agents reach the end of the network, their tags could be examined and then used to validate the path taken and determine the output result.

Here we provide formal semantics to NBC by defining transition relations that capture the dynamics of an agent in the network. This forms the basis of a translation into the SMV format supported by the NuSMV [9] and nuXMV [7] model checkers and its application to verify design correctness or identify logical errors. We also extend the NBC semantics to a real time stochastic model by mapping NBCs to chemical reaction networks (CRNs) opening up possibilities to utilize stochastic simulation and probabilistic model checking. Finally our formal model can serve as a starting point for computational complexity studies of the power and limitations of NBC systems.

## Related Work

Engineering biological devices to perform specified computation has the potential of utilizing the inherent parallelism in biological components to speed computation, construct low energy consuming devices and interface with biological material. Seminal work by Adelman [3] has demonstrated a method to use DNA for solving the Hamiltonian path problem, which is known to be NP-Complete. The instance of the Hamiltonian path considered in [3] is a small graph (7 nodes and 14 edges), thus a major challenge since then in the field is overcoming physical and experimental constraints towards scaling up the computation to tackle large systems.

There have been several different paradigms suggested to realize the vision proposed in [3], including DNA Strand Displacement Systems (DSD) [23, 25] that utilize the complementarity of DNA base sequences to bind together and perform designed reactions, and DNA self assembly applying a reprogrammable set of DNA tiles, capable of implementing a wide variety of self assembly algorithms [24, 28]. DNA walkers are molecular machines that can move along tracks [26, 30] and can be used for performing computation or moving cargo for nanotechnology applications. Computational methods and tools have proven to be useful in improving and validating the designs of engineered biological systems [5, 15, 22] and have served as motivating applications for defining semantics and computational methods for NBC. Formal verification methods assuming discrete semantics have been used to verify the correctness of DNA Strand Displacement Systems and DNA walkers [17, 31], and probabilistic model checking has also been applied to these systems [6, 11, 17]. More broadly, viewing biological systems as reactive systems leads naturally to specifying their behavior using temporal logic and applying model checking (see e.g. [8, 13] and references within).

Network-Based Biocomputation (NBC) [20] uses biological agents that operate in parallel to explore manufactured planar devices. To enable the exploration of the solution space effectively, NBC encodes the operations of solving NP-complete problems into graphs, which are then used as templates for the design and fabrication of networks, for instance microfluidic networks. To process the computation in a massively parallel fashion, NBC uses a large number of motile agents to explore the many possible paths towards actual solutions. The actual circuits we have verified here are physically manufactured to be populated with actin filaments or microtubules [4], although similar devices have been experimentally implemented for bacteria [27]. In [29], the SSP problem has been solved by the NBC approach using a laser photonic system rather than molecular motors as in [20]. Our computational methods and tools are applicable to all the variety of experimental implementation strategies currently developed for NBC and can also be extended to support future NBC technology.

## Formal Semantics

We first describe our general approach for providing semantics to NBC circuits, the definitions are then used and refined to encode specific designs to solve the subset sum (SSP), exact cover (ExCov) and satisfiability (SAT) problems. A network is composed of a set of junctions that are positioned on a 2-dimensional plane, allowing agents to move along the network to nearby junctions according to the type of junction visited. The encoding assumes a single agent traversing the network, and can naturally be used to construct a system consisting of several agents traversing the network in parallel. We define a discrete state semantics that includes nondeterministic choice, and then suggest a translation to chemical reaction networks (CRNs) [10] that provides a stochastic continuous time semantics.

### Discrete Nondeterministic Semantics

Our transition system is defined as:

$$ T = \langle V,\theta ,\rho , C)$$

Where *V* are the system variables, $\theta$ is the initial condition, $\rho$ is the transition relation and *C* is a set of compassion requirements. The variables encode the position of the agent in the network and its direction of movement:

$$ V = \{x, y, dir\}$$

The variables *x* and *y* encode the position of the agent in the network, where $x \in \{0 \cdots max \}$ and $y \in \{0 \cdots max \}$ and *max* is the sum of all elements in the set in the case of the subset sum problem, determining the size of the device in the general case. The variable *dir* is a Boolean variable encoding the direction of movement of the agent. In most circuits we assume the initial condition $\theta$ is $x = 0 \wedge y = 0$ capturing an agent entering the circuit from the upper left corner, see Fig. 1a. We assume here the initial position is a split junction and do not constrain the value of the *dir* variable, thus it can be chosen nondeterministically to be either 0 or 1. The variable *dir* maintains the current movement direction of the filament, where $dir = 0$ means travelling down while $dir = 1$ means travelling diagonally.

The transition relation specifies how the variables get updated depending on the current state:

$$y' = y + 1$$

$$(x' = x \wedge dir = 0) \vee (x' = x + 1 \wedge dir = 1)$$

Agents move from the top row to the bottom row, thus the *y* variable always gets incremented by 1 specifying this movement. The movement can either be directly down, in which case *x* is not changed, this happens when the variable *dir* is 0, or diagonally, in which case *x* is incremented by 1, when the variable *dir* is 1. In addition we update the transition relation such that after reaching the bottom row the agent returns back to the top left corner of the network, to the state $x = 0 \wedge y=0$.

The variable *dir* determines the direction of movement as explained above. It remains unchanged if the agent is in a pass junction, or makes a nondeterministic choice between 0 (down) or 1 (diagonal) if the agent is in a split junction:

$$dir' = (dir \wedge (x',y') \in pass) \vee (\{0,1\} \wedge (x',y') \in split)$$

We define the compassion requirement:

$$ C = \{\langle ( x = m \wedge y = n \wedge (m,n) \in split , x = m \wedge y = n+1) \rangle , $$

$$ \langle ( x = m \wedge y = n \wedge (m,n) \in split , x = m+1 \wedge y = n+1) \rangle \}$$

A compassion requirement is composed of a set of pairs, each pair is of the form $\langle p, q \rangle$ and requires that if *p* appears infinitely often then *q* appears infinitely often. In this case for every split junction if it is visited infinitely often it will take the direction down infinitely often and the direction diagonal infinitely often. This ensures that for every split junction both directions, down and diagonal will eventually be explored. Formally, if the state $x = m \wedge y = n$ that is a split junction is visited infinitely often, then both of the states $x = m \wedge y = n+1$ and $x = m + 1 \wedge y = n + 1$ will be visited infinitely often.

### Stochastic Semantics

Following from the semantics described above we propose a stochastic semantics extension by providing a mapping to chemical reaction networks (CRNs). CRNs consist of a set of species *C* and a set of reactions *R* that allow the species to interact. We introduce species for each of the locations in the network, with a separate species for down or diagonal movement if the position is a pass junction.

For split and pass junctions the species are, respectively:

$$ C_s = \{x_iy_j | i,j \in \{0 \cdots max \} \wedge (i,j) \in split \}$$

$$ C_p = \{x_iy_jd_k | k \in \{0,1\},i,j \in \{0 \cdots max \} \wedge (i,j) \in pass \}$$

The species will count how many agents are positioned at each location described by state $x=i \wedge y=j$, allowing to represent multiple agents simultaneously exploring the network. The reactions will correspond to an agent moving to the next location. For each split junction, assuming the next junction is a pass junction, we will define the following two reactions:

$$x_iy_j \rightarrow x_iy_{j+1}d_0$$

$$x_iy_j \rightarrow x_{i+1}y_{j+1}d_1$$

If an agent is in a split junction at position (*i*, *j*) there are two reactions as shown above that can be taken, the first will move the agent to position $(i,j+1)$ representing a down movement, whereas the second will move the agent to position $(i+1,j+1)$ representing a diagonal movement. If the first equation is fired then the number of copies of species $x_iy_j$ will be decremented by 1 and the number of copies of species $x_iy_{j+1}d_0$ will be incremented by 1, whereas if the second equation is fired, the number of copies of species $x_iy_j$ will be decremented by 1, and the number of copies of species $x_{i+1}y_{j+1}d_1$ will be incremented by 1.

For pass junctions, assuming the next junction is also a pass junction, we define the following reactions, in which according to the first reaction the movement is down and according to the second reaction the movement is diagonally:

$$x_iy_jd_0 \rightarrow x_{i}y_{j+1}d_0$$

$$x_iy_jd_1 \rightarrow x_{i+1}y_{j+1}d_1$$

If the next position is a split junction we define the following reactions:

$$x_iy_jd_0 \rightarrow x_{i}y_{j+1}$$

$$x_iy_jd_1 \rightarrow x_{i+1}y_{j+1}$$

**Fig. 2.**
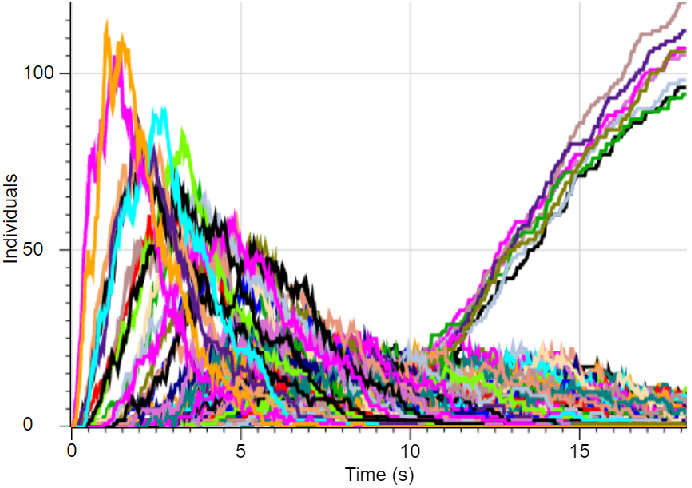
Stochastic simulation of an SSP network for $$S = \begin{Bmatrix} 2&5&9 \end{Bmatrix}$$ with 1000 agents. Time is shown in the *X* axis while the number of individual agents of each species is shown in the *Y* axis. Each color plot represents a different species at a specific network position. This simulation starts with 1000 individuals at position $(x,y)=(0,0)$ (plot not shown) that traverse the network assuming no interaction between the agents. The graph is a result of running a CRN model using Gillespie stochastic simulation implemented in the DSD tool [18]. The plots that rise beyond the background values at around 10 time units are the number of agents at each of the 8 possible subset sum exits.

The CRN defined above can also have a rate associated with each reaction which is a number that determines the probability of firing the reaction effecting how fast these reactions will fire. These definitions provide a stochastic continuous time semantics for NBCs using the underlying CRN model [10]. An example of a stochastic simulation using these semantics for the SSP network from Fig. 1a is shown in Fig. 2.

We next explain our encodings of the SSP, ExCov and SAT problems and the temporal logic properties used to specify the correctness of the circuits. Our motivation here is to capture the networks used in the experimental work with the actual biological agents and not to find efficient ways to solve these NP-Complete problems on standard computers. The verification approach can then be generalized and utilized to NBC applications in which the main aim is to interact with living cells for diagnostic and medical applications rather than solve combinatorial problems.

## Subset Sum Problem (SSP)

The Subset Sum Problem (SSP) is an established NP-Complete problem that returns *true* if a subset exists in a given set *S*, that sums to some given value *k*, and returns *false* otherwise.

The SSP network is constructed using variables for rows, columns, junction types, movement direction of computational agents, and a flag. The flag is used to indicate that the computational agent has reached the network output (the last row).

An additional tag variable was added to the network description in order to track at which split junctions the computational agents took a diagonal path, thus “adding” the element for that row. The tag is built by indexing split junctions starting from the top left corner of the network (index 0) and then running through each row and assigning indices to the junctions sequentially. This indexing includes junctions that are considered unreachable in a properly functioning network. Networks using tagging are able to identify the exact path taken to reach a given sum. This allows further investigation into the number of different paths to a given output. In experimentally manufactured NBC devices these tags may also allow for identification of agents that followed erroneous paths.

Agent positioning in the network is indicated by row and column variables that run from zero to the maximum sum of elements in the given set. Only half of these (*row*, *column*) points are used due to the network’s triangular structure. In order to define the transition relations for the general SSP problem, $$S = \begin{Bmatrix} s_1&s_2&\ldots&s_N\end{Bmatrix}$$, we first define the maximum sum of set *S* (Eq. 1), array of split junction rows (Eq. 2) and, if tagging is used, an array of tags (Eq. 3).

1 $$\begin{aligned} max=\sum _{i=1}^{N} s_{i}\end{aligned}$$

2 $$\begin{aligned} srow=\left[ 0 \sum _{i=1}^{index} s_{i}\right] \text{ where } {index}=1, \ldots , N-1\end{aligned}$$

3 $$\begin{aligned} tag=\left[ \begin{array}{llll} t_{0,0}&t_{\varSigma _{i=1}^{index}s_{i}, 0}&\dots&t_{\varSigma _{i=1}^{index} s_{i},\varSigma _{i=1}^{index} s_{i}} \end{array}\right] \text{ where } {index}=1, \ldots , N-1\end{aligned}$$

The row increases with each transition until reaching the end of the network. This captures the assumption that agents cannot move backwards in the network. Junction type, which depends on the row, is decided according to a sequential sum of elements in the set. The direction of movement is either nondeterministic (when “choosing” at a split junction) or keeps the last selection (when at a pass junction). The full transition relation, without the additional tag variable, can be seen in Eq. 4. The tag’s transitions are separately defined in Eq. 5.4
5A duplicate network was built with the addition of two variables, *sum* and *xsum*, for verification of overall output correctness, rather than specific output correctness. These variables select a value from the set of valid sums and the set of invalid sums respectively, and are used for comparison with the column value when reaching the network output.

**Table 1.**
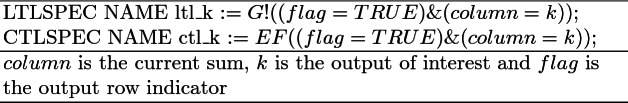
Network specifications for individual outputs. LTL specification (ltl_k) checks that the output of interest is never reachable. CTL specification (ctl_k) checks if there is any path to the output of interest.

Two specification types were used to verify network correctness. The first type (Table 1) uses both Linear Temporal Logic (LTL) and Computational Tree Logic (CTL) to check the validity of a specific sum *k* by comparing it with the column value at the output. The LTL formula checks the lack of a run where $column = k$, while the CTL formula checks for the existence of at least one run where $column = k$. For the SSP, the value *k* can range anywhere from zero to the maximum sum value of set *S*. We use both CTL and LTL although the outcomes of NBC verification will be equivalent, for evaluating and optimizing the performance of model-checking, as discussed in Sect. 7.

The second type of specification (Table 2) uses CTL to check that all valid sums are reachable and all invalid sums are unreachable. When used on networks containing identifiable errors (errors that can be detected by measuring agents at the exit of the network in the bottom row), a counter-example is provided indicating an unreachable valid sum and/or a reachable invalid sum. This specification does not need to get a target sum *k* but rather checks correctness for any target sum.

**Table 2.**
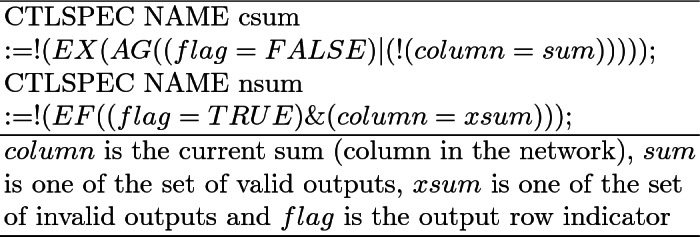
Network specifications for overall output in CTL. csum checks that the network can exit on all valid sums. nsum checks that the network cannot exit on any invalid sum.

## Exact Cover (ExCov)

The Exact Cover problem (ExCov) is another important problem, which is known to be NP-Complete. This problem returns *true* if there exists an exact cover (a union of disjoint sets) of the defined universe *U* when given a collection of sets *SS* that contain elements from the universe, and returns *false* otherwise.

We use a reduction to SSP to construct NBCs that solve the ExCov problem [16]. In the reduction, the ExCov is encoded into binary format. This encoding is then used to create the elements of an SSP network. The elements of the universe are treated as an array, where each position can be either 0 or 1, and where each element is given a specific index in the array. The sets to be examined are then each assigned an array of the same length as the universe, where only elements contained in the set are assigned a “one” value. All other elements are assigned a “zero” value. These arrays are then treated as binary numbers and are converted to their respective decimal values, as shown in Table 3.

**Table 3.**
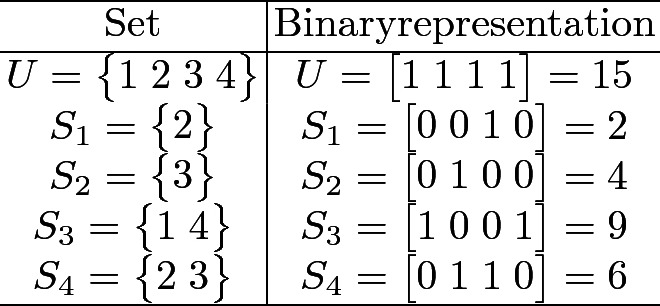
Conversion from set to decimal using binary representation.

As the ExCov does not allow the union of non-disjoint sets (the exact cover cannot contain sets that share an element), a “force-down” junction is included in the network to replace such split junctions. This prevents the agents from taking a diagonal path where an element in the current set is already contained in a previously included set on the path.

**Fig. 3.**
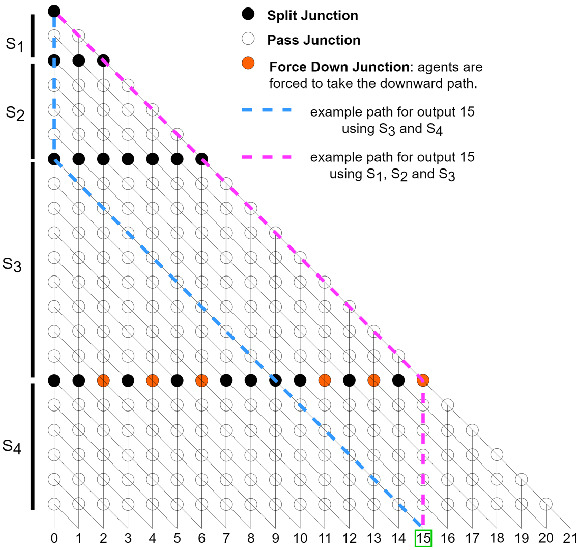
ExCov network for $$U = \begin{Bmatrix} 1&2&3&4 \end{Bmatrix}$$ and $$SS = \begin{Bmatrix}\begin{Bmatrix}2\end{Bmatrix},&\begin{Bmatrix}3\end{Bmatrix},&\begin{Bmatrix}1&4\end{Bmatrix},&\begin{Bmatrix}2&3\end{Bmatrix}\end{Bmatrix}$$. Split and pass junctions are as defined in Fig. 1a. Force-down junctions are denoted as filled orange circles. The blue path combines sets $S_3$ and $S_4$, constituting an exact cover. (Color figure online)

This construction can be seen in Fig. 3, which depicts the network for the sets given in Table 3. There exist multiple exact covers for this set of subsets, so there are multiple paths in this network that lead to output 15, the binary encoding of the universe. The pink path exhibits the function of the force-down junctions, where the computational agent is forced into the downward direction instead of having the chance to move diagonally, as in a split junction. In this case, this is due to set $S_4$ sharing elements with sets $S_1$ and $S_2$, which have already been included. In terms of the decision problem encoded in the network, the existence of one path leading to the required output implies that the result should be computed as *true*.

This network is, in essence, an implementation of the SSP network with the addition of a new junction type. Thus, the state of the model is defined by the same combination of variables as that of the SSP. The junction type now depends on both row and column values as the previously defined split junction rows may now contain force-down junctions. The tag variable was added here as well, to track the path taken by the biocomputation agents. The maximum sum of the network, split junction rows, and tags are defined as they were in SSP, where the set elements are now the decimal values of the subsets’ binary representation. The transition relation, without the additional tag variable, can be seen in Eq. 6, while the tag’s transitions are defined in the same manner as the tags for the SSP (Eq. 5).6Both LTL and CTL specifications were used to verify the output of interest *k*, similar to the specifications in Table 1. The difference here is that *k* is assigned the decimal value of the binary representation of the universe.

## Satisfiability (SAT)

The Boolean Satisfiability problem (SAT) is considered the classic NP-complete problem. SAT is the problem of determining if there exists an assignment of *true* and *false* values to the variables of a Boolean formula, such that the formula evaluates to *true*. The formula is considered satisfiable if any such assignment exists, and is considered unsatisfiable when no such assignment exists (the formula always evaluates to false). One standard format for SAT problems is Conjunctive Normal Form (CNF), where the Boolean formula $\varphi$, consists of a conjunction of a set of clauses $$\begin{Bmatrix}C_i\end{Bmatrix}^{n}_{i=1}$$, and each clause consists of a disjunction of a set of literals $$\begin{Bmatrix}x_j\end{Bmatrix}^{m}_{j=1}$$.

**Fig. 4.**
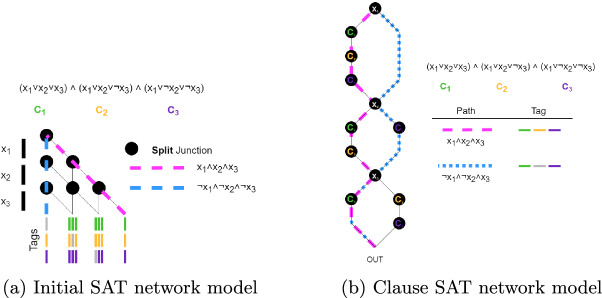
SAT network models for three literals and three clauses.

The initial model designed for SAT used a similar structure to that of the SSP network, as seen in Fig. 4a. Each row represents a literal $x_j$, and each junction is a split junction. As computational agents progress through this network, they are tagged after each split junction for the clauses their truth assignment satisfies. The two example paths demonstrate cases where all tags are marked (the Boolean formula was satisfied), as well as where there was a tag missing (the Boolean formula was not satisfied). As there exists an output where all tags are marked, the problem is satisfiable.

The next network model used, seen in Fig. 4b, is structured with individual junctions for literals and clauses, rather than having multiple junctions for each literal as in Fig. 4a. Each literal junction has paths both to the left (*true*) and right (*false*), reflecting their relevant truth assignment. These paths connect to a sequence of clause junctions. Computational agents are tagged at clause junctions with an identifier for the relevant clause satisfied by the truth assignment of the path.

Unlike the SSP and ExCov networks where the output location indicates the result, in the SAT network, the use of tagging is critical as it indicates the clauses satisfied. The final computation result depends on the total collection of tags on the computational agents at output. The problem is considered satisfiable if there exists an agent that collected tags for each clause as measured at output.

Using the clause model, two network descriptions were constructed. One network description has separate variables for clause junctions and tags, while the other unifies them into a single tag variable that merges their behavior in order to minimize the number of variables and possible states created by the NuSMV model checker. The tag variables for these networks are treated as counters that indicate the number of times each clause has been satisfied. As all problems investigated are of the 3-SAT format, the tag for each clause can only be an integer from zero to three, where zero indicates the clause was never satisfied.

The state of the model is defined by a combination of variables for junction type, direction of movement, current literal and it’s assigned value, exit flag, and a tag array for the clauses satisfied. The junction type is now divided into clause and literal junctions.

**Table 4.** SAT clause network LTL and CTL specifications. For satisfiable networks LTL returns *false* and CTL returns *true*. For unsatisfiable networks LTL returns *true* and CTL returns *false*.

LTL	LTLSPEC NAME ltl_sat $:= G! ((flag = TRUE) \& (\bigwedge \limits _{i\ge 0} tag[i] > 0))$
	There is no path that satisfies all clauses
CTL	CTLSPEC NAME ctl_sat $:= EF ((flag = TRUE) \& (\bigwedge \limits _{i\ge 0} tag[i] > 0))$
	There exists a path that satisfies all clauses
Each *tag*[*i*] corresponds to a specific clause	

Both models use the same LTL and CTL specifications to check if all tags have a positive, non-zero value when reaching the output state. That is, $tag[i] > 0$ for every clause *i* when $flag = TRUE$. The number of tags directly corresponds with the number of clauses.

## Experimental Results

We developed a prototype tool [1, 2] that automates both the generation of the SMV encodings for each problem (SSP, ExCov and SAT), and the verification of these encodings using the NuSMV model checker [9]. The user selects which problem they would like to solve and then the tool runs as described in the following sections. For the SSP and ExCov problems our tool also automates the translation to chemical reaction networks allowing to run a Gillespie stochastic simulation using the GEC and DSD tools [18, 21]. We systematically evaluate the verification capabilities of our tool, by proving correctness of the designs and by identifying errors in designs that were explicitly modified to represent faulty junctions or errors in the NBC encoding. Overall the verification results demonstrate that the approach can handle large NBC circuits and is applicable to the real-world systems currently designed in [4, 20, 27].

### SSP

Using input sets from the user, the tool builds SMV network descriptions both with and without tags. Once the models have been generated, the tool runs NuSMV on each of the defined specifications. Verifications are first run on the specifications defined in Table 1 using two methods. The first runs all outputs in bulk, and the second runs output by output. This is done for both LTL and CTL specifications separately. Then, verifications are run on the specifications defined in Table 2 for both valid and invalid sums. Each specification’s verification result and runtime is parsed and saved for further analysis.

**Table 5.** SSP all output verification runtimes in minutes

SSP						
ID	Set size	Set	Tag runtimes		No tag runtimes	
			LTL	CTL	LTL	CTL
0	3	[2, 3, 5]	0.0041	0.0016	0.0035	0.0014
1	4	[2, 3, 5, 7]	0.0114	0.0027	0.0073	0.0022
2	5	[2, 3, 5, 7, 11]	0.0478	0.0065	0.0198	0.0038
3	6	[2, 3, 5, 7, 11, 13]	0.2256	0.0218	0.0466	0.0070
4	7	[2, 3, 5, 7, 11, 13, 17]	1.3204	0.0956	0.1028	0.0138
5	8	[2, 3, 5, 7, 11, 13, 17, 19]	18.0535	0.4476	0.2144	0.0278
6	9	[2, 3, 5, 7, 11, 13, 17, 19, 23]	106.7040	2.0753	0.4226	0.0553

While the difference between LTL and CTL verification runtimes in small networks is negligible, the difference in large networks is considerable. As seen in Table 5, LTL runtimes grow at a much faster rate than those of CTL. There is also a drastic increase in runtime when verifying networks utilizing tagging, as additional variables are necessary to define tags for all split junctions. For the first specification type, it is not usually necessary to look at all outputs or both logics. Thus, runtime can be decreased by examining specific outputs of interest using a single specification instead. The increase in verification runtime as a result of larger network size is not as drastic for running individual outputs (Table 6) due to the compounded nature of the runtime when running in bulk.

**Table 6.** SSP output 9 and 10 verification runtimes in minutes.

SSP								
ID	Set size	Set	Output	Path exists	Tag runtimes		No tag runtimes	
					LTL	CTL	LTL	CTL
0	3	[2, 3, 5]	9	NO	0.0012	0.0010	0.0011	0.0010
0	3	[2, 3, 5]	10	YES	0.0015	0.0010	0.0013	0.0009
1	4	[2, 3, 5, 7]	9	YES	0.0020	0.0010	0.0015	0.0009
1	4	[2, 3, 5, 7]	10	YES	0.0020	0.0010	0.0015	0.0009
2	5	[2, 3, 5, 7, 11]	9	YES	0.0033	0.0012	0.0019	0.0010
2	5	[2, 3, 5, 7, 11]	10	YES	0.0033	0.0012	0.0019	0.0010
3	6	[2, 3, 5, 7, 11, 13]	9	YES	0.0082	0.0019	0.0023	0.0011
3	6	[2, 3, 5, 7, 11, 13]	10	YES	0.0083	0.0018	0.0023	0.0011
4	7	[2, 3, 5, 7, 11, 13, 17]	9	YES	0.0278	0.0032	0.0030	0.0012
4	7	[2, 3, 5, 7, 11, 13, 17]	10	YES	0.0281	0.0033	0.0030	0.0012
5	8	[2, 3, 5, 7, 11, 13, 17, 19]	9	YES	0.2507	0.0079	0.0041	0.0013
5	8	[2, 3, 5, 7, 11, 13, 17, 19]	10	YES	0.2510	0.0079	0.0041	0.0014
6	9	[2, 3, 5, 7, 11, 13, 17, 19, 23]	9	YES	1.1600	0.0306	0.0057	0.0015
6	9	[2, 3, 5, 7, 11, 13, 17, 19, 23]	10	YES	1.1433	0.0245	0.0057	0.0015

Verification runtime for the second specification type grows at about the same rate as that of the bulk run on the first specification’s CTL format (Table 7 and Table 8). The two are comparable as they both check validity of all network outputs. By using these different specification types, we are able to efficiently verify NBC designs for increasingly large networks.

**Table 7.** SSP general sum verification runtimes in minutes.

SSP					
Set size	Set	Runtime			
		csum		nsum	
		Tag	No Tag	Tag	No Tag
3	[2, 3, 5]	0.0011	0.0011	0.0009	0.0009
4	[2, 3, 5, 7]	0.0013	0.0012	0.0009	0.0009
5	[2, 3, 5, 7, 11]	0.0018	0.0013	0.0009	0.0009
6	[2, 3, 5, 7, 11, 13]	0.0037	0.0018	0.0009	0.0009
7	[2, 3, 5, 7, 11, 13, 17]	0.0092	0.0025	0.0009	0.0009
8	[2, 3, 5, 7, 11, 13, 17, 19]	0.0260	0.0042	0.0009	0.0009
9	[2, 3, 5, 7, 11, 13, 17, 19, 23]	0.0821	0.0074	0.0010	0.0009

**Table 8.** SSP general sum verification runtimes in minutes on network with no tag variable. Sets include the first *k* prime numbers.

SSP				
Set size	Set	Runtime		
		csum	nsum	Total
5	[2, 3, 5, 7, 11]	0.0055	0.0013	0.0068
10	[2, 3, 5, ... 19, 23, 29]	0.1140	0.0025	0.1165
15	[2, 3, 5, ... 41, 43, 47]	1.5203	0.0024	1.5227
20	[2, 3, 5, ... 61, 67, 71]	7.8919	0.0036	7.8955
25	[2, 3, 5, ... 83, 89, 97]	32.3312	0.0059	32.3371
30	[2, 3, 5, ... 107, 109, 113]	122.3742	0.0112	122.3854

The second specification type can further be used to identify unreachable valid sums and reachable invalid sums in networks with observable errors. We model here errors that my occur as part of the manufacturing of the NBC devices, and consider a scenario where a certain junction appears to contain an error and we want to check its effect on the correctness of the overall circuit. There are three general types of errors that may be found in SSP networks:

1. Pass junction behaves as a split junction
2. Pass junction forces one direction
  - when both paths are valid (block one valid path)
  - when one path is valid, and the invalid path is forced
3. Split junction forces one direction

**Fig. 5.**
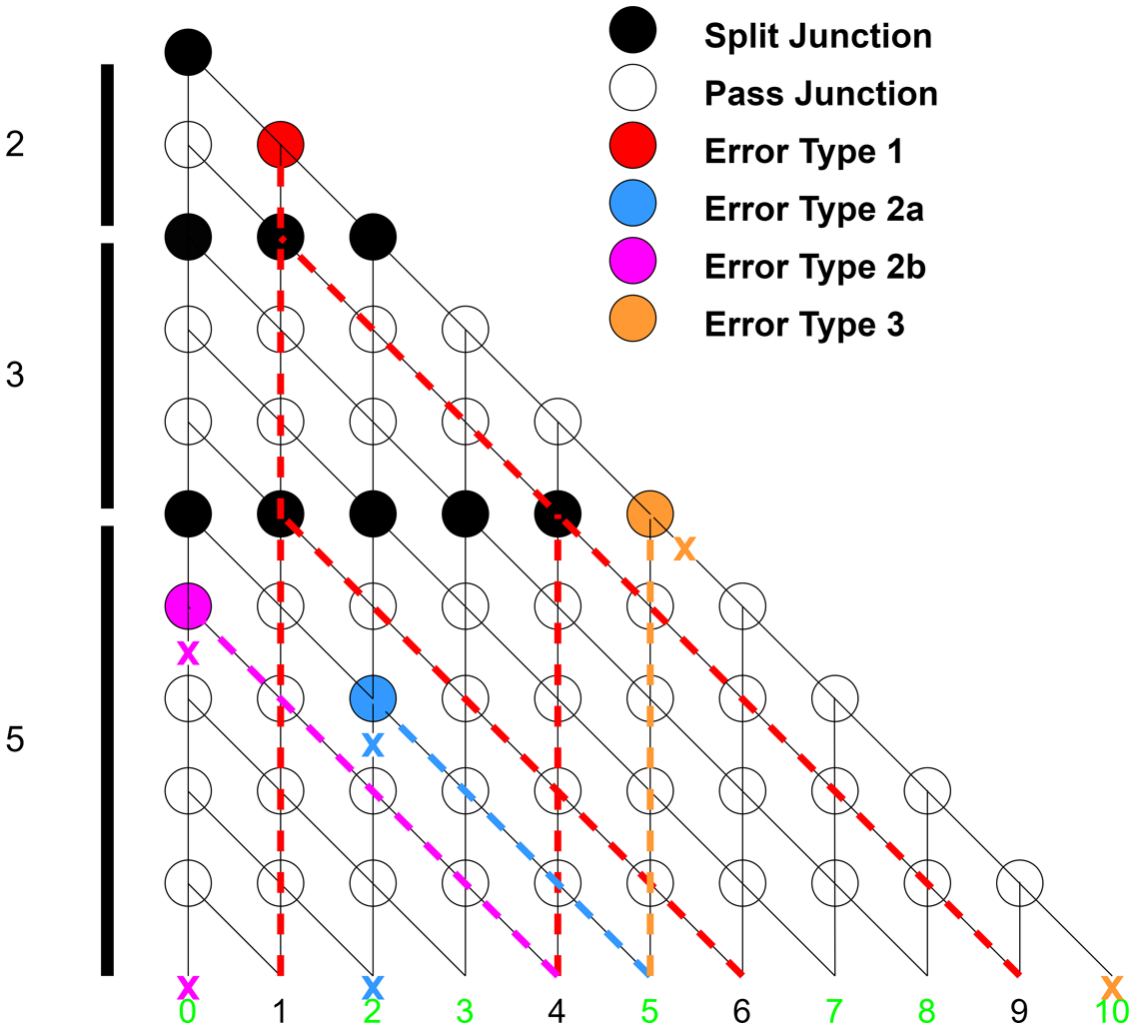
SSP network for $$S = \begin{Bmatrix} 2&3&5 \end{Bmatrix}$$ with example errors and their resulting outputs. Each error type is assigned a color. Resulting reachable paths are marked with dashed lines. Blocked paths are marked with an X at the initial and end points.

Examples of these errors are shown in Fig. 5. These errors are not always identifiable by observing the possible exits from the network, as affected junctions may not be reachable, forced paths may converge with valid paths, or blocked paths may not be the only path leading to the affected output. In order to simulate manufacturing errors that would cause unexpected outputs, deliberate errors were added to the network descriptions. A comparison between the expected verification result of the network and that of the network with added errors is shown in Table 9. The correctness of NBC network design can be checked by examining these errors and their verification results.

**Table 9.** SSP general sum verification results for valid networks and observably invalid networks. Error is denoted as the (*row*, *column*) junction location along with the error type as in the error type definitions.

SSP						
Set size	Set	Original network		Faulty network		
		csum	nsum	Error	csum	nsum
3	[2, 3, 5]	VALID	VALID	(3,1) - 2b	INVALID	INVALID
4	[2, 3, 5, 7]	VALID	VALID	(12,2) - 2a	INVALID	VALID
5	[2, 3, 5, 7, 11]	VALID	VALID	(14,4) - 1	VALID	INVALID
6	[2, 3, 5, 7, 11, 13]	VALID	VALID	(17,17) - 3	INVALID	VALID
7	[2, 3, 5, 7, 11, 13, 17]	VALID	VALID	(29,15) - 2a	VALID	VALID

### ExCov

Using an input file containing a collection of universes and sets of subsets, the tool encodes the given problems into binary format. Then, tagged and not tagged networks are generated using specifications for the output of interest as defined in Table 1. In this case, the output of interest (decimal value of universe’s binary encoding) is assigned to variable *k*. Then, NuSMV is run on both specifications (LTL and CTL) to check for the existence of an exact cover. The tool then parses and saves verification results and runtimes for further analysis.

Verification runtimes show similar behavior to those seen with SSP networks. The same difference in growth in runtime of LTL and CTL, as well as the same drastic difference in runtime of tagged as compared to not tagged networks is observed (Table 10).

**Table 10.** ExCov verification runtimes in minutes.

ExCov								
ID	Universe	# of Subsets	Set of subsets	ExCov exists	Tag runtimes		No tag runtimes	
					LTL	CTL	LTL	CTL
0	[1, 2, 3, 4]	4	[[1, 2], [1], [1, 3], [4]]	NO	0.0015	0.0013	0.0012	0.0011
1	[1, 2, 3, 4]	4	[[1, 2], [1, 3], [1, 3, 4], [1, 2, 3]]	NO	0.0016	0.0015	0.0014	0.0011
2	[1, 2, 3, 4]	4	[[2], [3], [1, 4], [2, 3]]	YES	0.0020	0.0009	0.0017	0.0010
3	[1, 2, 3, 4, 5, 6, 7, 8]	8	[[1, 4, 7], [1, 4], [4, 5, 7], [3, 5, 6], [2, 3, 6, 7], [2, 7], [8], [3, 4, 5]]	YES	666.1414	3.7020	0.0586	0.0079
4	[1, 2, 3, 4, 5, 6, 7, 8]	8	[[1, 4, 7], [1, 4], [4, 5, 7], [3, 5, 6], [2, 3, 6, 7], [2, 7], [4, 8], [3, 4, 5]]	NO	5.1313	6.1802	0.0113	0.0056

As the ExCov NBC design is based off of that of the SSP, the types of errors observed in the SSP may occur here as well. As the translation is more complex due to the addition of “force-down” junctions, it is critical to make sure these junctions are added at all relevant locations. By not including these junctions in the network description properly, incorrect results may be observed when verifying the existence of an exact cover. As the network grows larger, it becomes more difficult to identify such errors. In order to capture such mistakes in network translation, an additional variable was used to switch junction behavior to that of split junctions, in essence switching the network with the SSP equivalent. This type of error does not affect networks where an exact cover exists as the original path to the universe output is not blocked. A comparison of network behavior in both cases is seen in Table 11. This illustrates the utility of the verification method to verify new NBC designs that are complex or include various network optimizations, and may have subtle design errors.

**Table 11.** ExCov existence verification on networks with properly functioning force-down junctions (Valid) and networks with force-down junctions that behave as split junctions (Invalid).

ExCov				
Universe	Set of subsets	ExCov exists	ExCov found	
			Valid	Invalid
[1, 2, 3, 4]	[[1, 2], [1], [1, 3], [4]]	NO	NO	NO
[1, 2, 3, 4]	[[1, 2], [1, 3], [1, 3, 4], [1, 2, 3]]	NO	NO	YES
[1, 2, 3, 4]	[[2], [3], [1, 4], [2, 3]]	YES	YES	YES
[1, 2, 3, 4, 5, 6, 7, 8]	[[1, 4, 7], [1, 4], [4, 5, 7], [3, 5, 6],	NO	NO	YES
	[2, 3, 6, 7], [2, 7], [4, 8], [3, 4, 5]]			

### SAT

Our tool generates 3-CNF SAT problems of random sizes in DIMACS format using CNFGen [19]. These are then run through the MiniSat SAT Solver to get their satisfiability results [12] for later comparison with NuSMV verification results. The tool then generates two network descriptions for each problem, one with separate clause and tag variables (Clause) and one with merged clause and tag variables (No-Clause). NuSMV is then run on each network description, once with and once without variable re-ordering (VRO). The re-ordering organizes the tag variables by first appearance of the relevant clause in the network. For example, all clauses containing the first literal come before clauses containing the second literal. Verification results and runtimes, for each of the specifications defined in Table 4, are parsed and saved for further analysis. NBC verification results were compared with the MiniSat results, which directly check satisfiability or unsatisfiability of the formula, and were all consistent.

Runtimes are examined using three comparisons; LTL vs. CTL, No VRO vs. VRO and No-Clause vs. Clause (Table 12). The same differences in verification runtime of LTL as compared with CTL specifications seen in SSP and ExCov were observed. While variable re-ordering may improve verification runtime, the re-ordering used here did not generally show improvement for all networks, and no tendency towards either improvement or deterioration was observed. Overall, the No-Clause network description tends to have faster runtimes than the Clause network description, as unification of the tag and clause variable decreases the size of the network description.

**Table 12.** SAT verification runtimes in minutes.

3-SAT									
# Clauses	# Variables	No-clause runtimes				Clause runtimes			
		No VRO		VRO		No VRO		VRO	
		LTL	CTL	LTL	CTL	LTL	CTL	LTL	CTL
18	31	6.8871	0.1432	100.0822	0.8676	59.2266	0.2711	86.9653	0.5320
19	38	25.7469	0.4413	103.6992	0.3943	369.7850	1.1077	76.2565	0.2613
14	26	5.8769	0.1064	56.8184	0.3577	20.1059	0.1606	13.9620	0.0781
9	23	0.0201	0.0017	0.0959	0.0039	0.0299	0.0022	0.0670	0.0032
15	37	0.4292	0.0138	12.7095	0.0370	11.8577	0.0233	10.9242	0.0284
13	32	0.0334	0.0033	1.7767	0.0104	0.5014	0.0051	1.8381	0.0094
19	27	37.3025	1.0872	194.4493	2.7105	348.9075	3.5467	123.0635	1.2737
10	27	0.0320	0.0025	0.0820	0.0045	0.0548	0.0029	0.0624	0.0032
19	19	1.6001	0.0982	0.1200	0.0106	12.7730	0.2503	1.8869	0.0288
3	9	0.0085	0.0013	0.0090	0.0014	0.0048	0.0012	0.0054	0.0012

## Summary

We presented a prototype verification tool that takes as input an NBC design and performs formal verification to check the logical correctness of the circuit. The tool verifies the correctness of NBC designs for SSP, ExCov and SAT. For handling SAT problems, we have also implemented tagging in the verification tool, where the agent sets all the labels it gathers while traversing the network to true, and temporal logic queries can also relate to the tagging of the filament when exiting the network. We have used our tool to analyze the efficiency of different methods of verifying encodings and to generate random examples of varying sizes and difficulties using an automatic SAT formula generator. The verification results demonstrate that the approach can handle large NBC circuits and is applicable to the real-world systems currently designed in [4, 20, 27]. Our work is currently used as an integral part of the design phases of new circuits in the Bio4Comp project.

Future work includes further scaling of the methods by evaluating and optimizing additional model checking algorithms and tools. Our translation to chemical reactions can form a basis for applying probabilistic model checking, which can remove some of the restricting assumptions made here. For example, we assume that pass junctions that do not have a manufacturing fault, never allow computational agents to change direction, while it was observed [4, 20] that most but not all of the of the agents traverse through pass junctions correctly. The effects of these errors could be quantified and analyzed using simulation and probabilistic model checking of CRNs to quantitatively estimate the effects of these errors in NBCs.
